# *Edwardsiella tarda* Infection Triggering Acute Relapse in Pediatric Crohn's Disease

**DOI:** 10.1155/2019/2094372

**Published:** 2019-03-20

**Authors:** A. K. Li, M. Barton, J. A. Delport, D. Ashok

**Affiliations:** ^1^Department of Pediatrics, Western University, London, Ontario, Canada N6A 3K7; ^2^Pathology and Laboratory Medicine, London Health Sciences Centre, London, Ontario, Canada N6A 5W9

## Abstract

Crohn's disease exacerbations can often be associated with bacterial infections causing gastroenteritis. We report a child who experienced exacerbation of his Crohn's disease associated with a positive stool culture for *Edwardsiella tarda (E. tarda)*. Endoscopy showed features of moderate inflammation similar to exacerbation of Crohn's disease. The patient was treated simultaneously with intravenous steroids and antibiotics, and his symptoms resolved. This case report highlights the importance of clinicians being able to promptly recognize and treat concurrent bacterial infections in Crohn's disease exacerbations.

## 1. Introduction

Crohn's disease (CD) is a lifelong chronic inflammatory bowel disease (IBD) that affects any part of the gastrointestinal (GI) tract. There are numerous pharmacological agents available to treat CD and achieve disease remission: steroids, immune modulators, and biologic therapies. Despite optimal immune-suppressive therapy, exacerbations of CD can occur. These are typically treated with higher doses of the aforementioned therapies, but they carry the risk of opportunistic pathogens overwhelming the patient's compromised immune system. CD exacerbations can also be mimicked or caused by bacterial pathogens, most commonly *Clostridium difficile* [1]. We report the first pediatric patient with CD exacerbation secondary to *E. tarda* infection, a rare human pathogen.

## 2. Case Report

An 8-year-old Caucasian boy presented in September 2013 with a history of frequent loose stools and bleeding per rectum. He was diagnosed with CD and treated with mesalamine (500 mg t.i.d.) initially. Following a CD exacerbation in October 2014, he was treated with oral prednisone regimen (40 mg daily tapering course) and azathioprine (50 mg oral daily). In March 2015, infliximab (biologic) therapy (200 mg IV) was initiated to optimize disease control. In May 2015, the patient was admitted to our institution for exacerbation of CD and *C. difficile* colitis. He was treated with vancomycin (350 mg oral q.i.d.) and metronidazole (400 mg IVq8h) for *C. difficile* infection.

The patient developed another CD flare up 3 months later and was reviewed urgently in our outpatient clinic. He was experiencing abdominal pain daily and approximately 7 loose, bloody bowel movements per day and weight loss. The patient most likely ingested contaminated fish prior to the onset of illness, but his close family members were well. No history of recent foreign travel. The patient was treated empirically for a possible recurrence of *C. difficile* infection with a 10-day course of vancomycin (375 mg oral q.i.d.) and metronidazole (250 mg oral t.i.d.). A repeat stool culture and *C. difficile* toxin was ordered at this visit.

**Figure 1:**
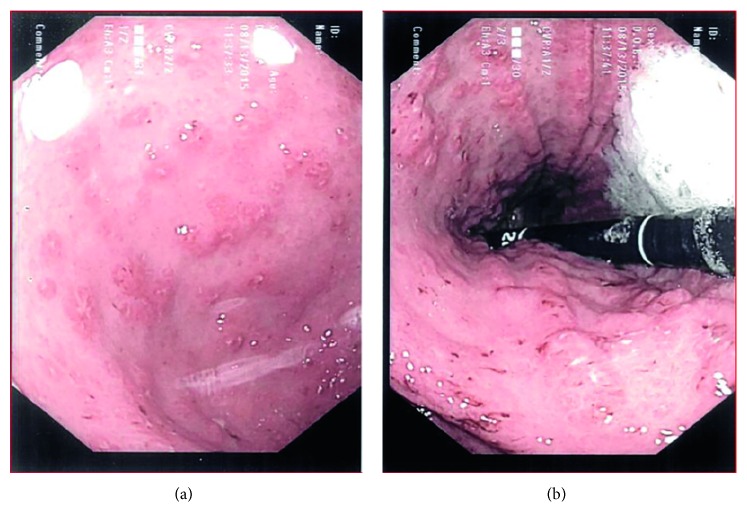
Upper endoscopy images: the stomach demonstrates multiple aphthous ulcerations, nodularity, friability, and erythema (typical of Crohn's).

**Figure 2:**
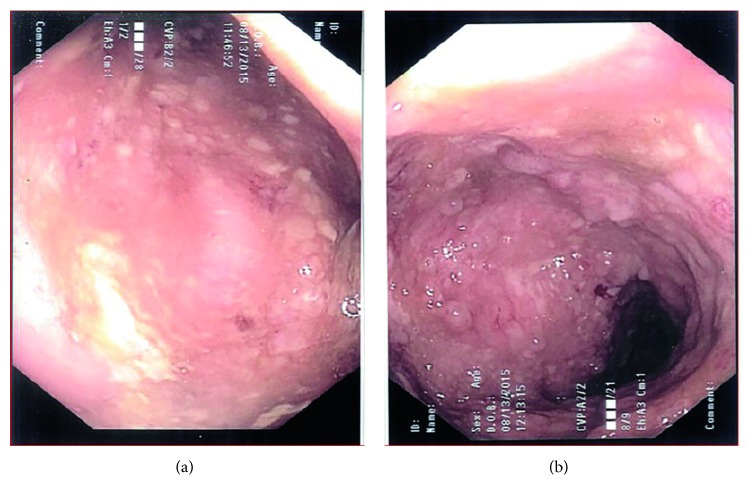
Lower endoscopy images: the colon shows diffuse moderate inflammation with ulcerations, mucosal edema, friability, cobble-stoning, and pseudopolyp formation indistinguishable from Crohn's disease.

Three days into treatment for *C. difficile*, his clinical status was getting worse. He had nausea, intermittent nonbilious vomiting, loose bloody stools, and poor appetite. He lost 8 pounds (10% weight loss) with significant lethargy. The worsening clinical status resulted in a hospital admission at his local hospital. The patient was started on methylprednisolone (40 mg IV daily) for a CD flare up and continued on vancomycin and metronidazole. It was subsequently determined that he had a positive stool culture for *E. tarda* but no evidence of *C. difficile.* He continued to be unwell with a fever (temperature 38.2°C), loose stools, and tachycardia (HR 112). He had mild diffuse tenderness in the lower quadrants. In view of his immune-compromised state and risk of bacterial translocation which may set him up for bacteremia, we transferred him to our tertiary centre. He was reviewed by the Pediatric Infectious Diseases team, and blood cultures were drawn prior to commencing intravenous ampicillin (1250 mg IV q6 hours). Therapy for *C. difficile* was continued as was steroid therapy. His blood investigations confirmed leukocytosis (25.5 × 10^9^ L) with neutrophilia (18.7 × 10^9^ L) and thrombocytosis (746 × 10^9^ L). The CRP was mildly elevated (16 mg L). Ultrasound imaging did not reveal any intra-abdominal abscesses. Endoscopy (Figures 1 and 2) and histopathology showed evidence of chronic active gastritis and colitis. Once blood cultures were negative, he was switched to oral amoxicillin (1000 mg t.i.d.). He had gradual clinical improvement and was discharged on a tapering regimen of prednisone 12 days later. Repeat stool culture was negative.

## 3. Discussion

There is a global rise in the incidence of inflammatory bowel diseases (IBDs). The etiology of IBD is not well understood; however, it has been postulated that infectious gastroenteritis may be a precipitating or exacerbating factor [2]. CD patients have been found to have higher concentrations of proinflammatory bacteria like bacteroides, eubacteria, and peptostreptococcus and reduced concentrations of protective bifidobacterial [3]. Synchronous enteric infections occur in 10.5–20% of IBD relapses [4]. Immunosuppressed patients with suspected CD flare who are only treated for the exacerbation run the risk of invasive infections with opportunistic bacteria. Since there may be a delay between stool collection and microbiology results, it is important for clinicians to always consider the possibility of concurrent bacterial infections and institute appropriate therapy promptly.

*E. tarda* is a Gram-negative, facultative anaerobic, rod-shaped bacterium that belongs to the Enterobacteriaceae family. *E. tarda* is isolated from fresh or brackish water and can also be found in reptiles, eels, or catfishes [5]. Turtles and snakes also act as reservoirs. Humans acquire the infection through exposure to fresh water and marine environments containing the bacteria or by eating raw fish [6]. While *E. tarda* is relatively rare, occurring in less than 5% of people, it can cause invasive infections like gastroenteritis, soft tissue infections, septicemia, hepatobiliary infections, meningitis, peritonitis, osteomyelitis, endocarditis, tubo-ovarian abscess, and salpingitis with significant mortality (22.7%) [7]. The most common presentation is gastroenteritis of varying severity with nausea, vomiting, abdominal cramping, fever, bloody diarrhea, colonic ulcerations, and pseudomembranes [6–8]. The mortality rate of *E tarda* infection is high in immune-compromised hosts (about 50%) and should be treated as a life-threatening infection [6]. The organism is susceptible to beta-lactams such as ampicillin, cephalosporins, and tazobactam as well as quinolones, nitrofurantoin, and gentamicin [9].

To our knowledge, this is the second documented case of a CD flare with *E. tarda* [10] and the first in a pediatric patient. We successfully treated our patient with IV Ampicillin and oral amoxicillin with complete recovery. This report underscores the value of performing a rigorous stool culture in all patients presenting with exacerbations of IBD.

## Abbreviations

CD: Crohn's disease
GI: Gastrointestinal.
